# Age-related changes in plasma extracellular vesicles influence neuroinflammation in the brain and neurological outcome after traumatic spinal cord injury

**DOI:** 10.21203/rs.3.rs-2821858/v1

**Published:** 2023-04-17

**Authors:** Zhuofan Lei, Balaji Krishnamachary, Rodney M. Ritzel, Niaz Z. Khan, Yun Li, Hui Li, Kavitha Brunner, Alan I. Faden, Junfang Wu

**Affiliations:** Department of Anesthesiology and Center for Shock, Trauma and Anesthesiology Research (STAR), University of Maryland School of Medicine, Baltimore, MD 21201, USA

**Keywords:** Spinal cord injury, old age, brain, inflammation, extracellular vesicles, microRNA

## Abstract

Approximately 20% of all spinal cord injuries (SCI) occur in persons aged 65 years or older. Longitudinal, population-based studies showed that SCI is a risk factor for dementia. However, little research has addressed the potential mechanisms of SCI-mediated neurological impairment in the elderly. We compared young adult and aged C57BL/6 male mice subjected to contusion SCI, using a battery of neurobehavioral tests. Locomotor function showed greater impairment in aged mice, which was correlated with reduced, spared spinal cord white matter and increased lesion volume. At 2 months post-injury, aged mice displayed worse performance in cognitive and depressive-like behavioral tests. Transcriptomic analysis identified activated microglia and dysregulated autophagy as the most significantly altered pathways by both age and injury. Flow cytometry demonstrated increased myeloid and lymphocyte infiltration at both the injury site and brain of aged mice. SCI in aged mice was associated with altered microglial function and dysregulated autophagy involving both microglia and brain neurons. Altered plasma extracellular vesicles (EVs) responses were found in aged mice after acute SCI. EV-microRNA cargos were also significantly altered by aging and injury, which were associated with neuroinflammation and autophagy dysfunction. In cultured microglia, astrocytes, and neurons, plasma EVs from aged SCI mice, at a lower concentration comparable to those of young adult SCI mice, induced the secretion of pro-inflammatory cytokines CXCL2 and IL-6, and increased caspase3 expression. Together, these findings suggest that age alters the EVs pro-inflammatory response to SCI, potentially contributing to worse neuropathological and functional outcomes.

## Introduction

The incidence of traumatic spinal cord injury (SCI) in the elderly has grown rapidly, with over 20% cases occurring in those over 65 years of age [20]. Although aging is a key risk factor for dementia, clinical evidence, including large-scale longitudinal population-based studies [10, 19, 39, 67], shows a higher incidence of cognitive decline, neurodegenerative disorders, and psychological/somatic comorbidities after SCI [8, 10, 55, 56]. Clinical magnetic resonance imaging studies reveal neuropathological changes in the brain following SCI [29]. Chronic neuroinflammation, decreased hippocampal neurogenesis, and increased neuronal endoplasmic reticulum (ER) stress have been reported after SCI in the brains of young adult mice and rats [2, 21, 23, 33, 38, 40, 63, 65, 68]. These were associated with posttraumatic hyperesthesia, impaired cognition and depression-like behavior. However, few studies have examined mechanistically how SCI affects the brain in young adult versus aged subjects. Such information is needed for the design of targeted interventions to limit the risk of cognitive decline or depression after SCI.

Clinical trials report that targeting inflammation improves mood and pain after SCI [1, 2]. We and others have shown that neuropsychological impairment after SCI is caused, at least in part, by microglial-mediated chronic inflammation in the brain [2, 21, 23, 33, 38, 40, 63, 65, 68]. Aging microglia show altered physiology, with evidence of cellular senescence and immune dysfunction [51]. We recently reported that a sub-population of microglia in older mice adopted a unique dysfunctional phenotype defined by changes in phagocytosis, oxidative stress, lysosomal content and autophagy, lipid and iron accumulation, metabolic alterations, pro-inflammatory cytokine production, and senescent-like features [49, 50]. However, whether age-related alterations in microglial and immune function are exacerbated in the brain after SCI is unknown.

The autophagy-lysosomal pathway is essential for intracellular lipid, protein, and organelle degradation and quality control [41]. We have previously shown that autophagy is dysregulated in injured CNS following trauma [34]. More recently, we demonstrated in both SCI and traumatic brain injury (TBI) models that perturbation of autophagy alters inflammatory responses: high levels of autophagy flux are associated with anti-inflammatory responses, whereas low levels promote pro-inflammatory phenotypes [17, 30]. Thus, inhibition of autophagy-lysosomal function could contribute to both neuronal cell damage and neuroinflammation observed in aging brain. Recent data [7] demonstrate an age-dependent decline in expression of autophagy genes [35, 37] and decreased lysosomal function. Although autophagic mechanisms have been found to decrease with age in many experimental models [36], how they are altered in the aging brain after SCI is unknown.

Among potential mechanisms leading to pathological changes in brain after SCI are changes in circulating extracellular vesicles (EVs) [12]. EVs contain proteins, lipids, microRNAs (miRs), and membrane receptors from their originating cells, which when delivered to recipient cells can alter their function and contribute to both physiological and pathophysiological processes [61]. Thus, it is plausible that EVs may contribute to secondary injury after SCI, not only locally but also systemically and in the brain. We recently characterized plasma EVs dynamics at acute and subacute time-points after SCI in young adult mice and showed that EVs derived from SCI mice can cause brain inflammation [22]. However, changes in plasma- and tissue-derived EVs in old age are not well understood.

In the present study, we assessed behavioral and histopathological changes in both young and aged mice after SCI. We analyzed the post-injury transcriptomic changes in the somatosensory cortex and hippocampus, which were further validated by flow cytometry. In addition, plasma EVs derived from young and aged mice following SCI were characterized with regard to their miR content and effects on microglia, astrocytes, and neurons *in vitro*.

## Materials and Methods

### Animals and mouse spinal cord contusion model

All animal experiments and surgical procedures were approved by the Institutional Animal Care and Use Committee (IACUC) at the University of Maryland School of Medicine. Young adult (10–12 weeks, 2.5–3.0-month-old) and aged (18-month-old) male C57BL/6 mice were obtained from Charles River Laboratories and housed on a 12 hours (h) light/dark cycle with food and water ad libitum. Moderate spinal cord contusion injury was conducted using the Infinite Horizon spinal cord impactor (Precision Systems and Instrumentation) as previously described [31, 64]. Briefly, the T10 segment of mouse spinal cord was exposed by laminectomy under anesthetization with isoflurane. The spinal column was stabilized with bilateral steel clamps and a midline spinal contusion at T10 level with a force of 60 kilodyne (kdyn) was conducted. Following surgeries, the bladders of injured mice were manually voided 2–3 times daily until a reflex bladder was established. Sham mice underwent the same procedure except for laminectomy and contusion. After surgeries, mice were randomly assigned to the groups based on their age. To minimize stress and fatigue, each behavioral test was performed on a different day.

### Assessment of hindlimb locomotion

Basso mouse scale (BMS) and the BMS subscore were recorded on day 1, day 3, and weekly thereafter for up to 49 days post-injury. The testing mouse was placed in a rectangular plastic open field (62 × 42 cm dimensions) with flat and smooth floor for 4-minute continuous observation by two trained researchers blinded to its group information. Animals were rated in a scale of 0 to 9 based on the standards as described previously [4].

### Y-maze test

This test was performed 1 day before SCI (baseline) and on day 49 post-injury. The Y-maze instrument (Stoelting, Wood Dale, IL) consisted of three identical arms (35 cm long, 5 cm wide, and 10 cm high) at an angle of 120° with respect to each other [50]. The distal 30 cm of each arm was defined as arm A, B, C. The rest was defined as the center. The mouse was placed at the end of a randomly picked arm facing the center and allowed to explore the maze freely for 6 minutes. An arm entry was defined as all four paws of the mouse entered the arm, and an alternation was designated when the mouse entered the three arms consecutively. The percentage of alternation was calculated as follows: total alternations × 100 / (total arm entries - 2). An arm return was defined as the mouse return to the same arm after entry to a different one. Its calculation is the same as the percentage of alternation. All the tests were videorecorded and analyzed by self-made software.

### Novel object recognition (NOR) test

The NOR, social recognition (SR), and novelty suppressed feeding (NSF) tests were conducted consecutively during 50 to 54 days post-injury. A Plexiglas open field with a 40 cm × 40 cm square gray floor and four of 35 cm high black walls were placed in a dark room with red light on [50]. Mice were placed in the center and allowed to move freely for 5-minute habituation on the first day. Next day, two identical objects were placed on the diagonal line symmetrical to the center, which were at a distance of 12–13 cm from the close walls. One of the familiar objects were randomly replaced with a novel object on the third day for each testing mouse. The time mice spent with two objects was recorded using ANY-maze software (Stoelting) until a sum total of 30 s exploration time was reached.

### Social recognition (SR) test

The SR test was performed using a three-chambered rectangular apparatus (60 × 40 × 23 cm) made of transparent Plexiglas [49]. It was divided into three equal chambers by two walls that each had a semicircular hole of 5 cm diameter in the bottom for free access to each chamber. Two identical wire mesh cylinder cups were placed in the corners of two side chambers separately for every single session. Each mouse was singly housed overnight then placed in the middle chamber for 3 minutes with the holes blocked by transparent partitions, which was followed by 10-minute free exploring with the partitions removed. Next day, a stranger mouse was introduced and randomly placed inside one of the empty cups. The testing mouse started from the middle chamber with the holes blocked and then freely explore all three chambers for 10 minutes with the partitions removed. Then, the testing mouse was led back to the middle chamber and the holes were blocked. A second novel stranger mouse was placed inside the other empty cup. After partitions removal, the testing mouse was once again allowed to freely explore all three chambers for another 10 minutes. All behaviors of the subject mice in the chambers were recorded and analyzed using ANY-maze software (Stoelting).

### Novelty suppressed feeding (NSF) test

All mice were weighed and singly housed, then underwent food deprivation for 24 hours before the test. A Plexiglas open field with a 40 cm × 40 cm square gray floor and four 35 cm high transparent walls was placed in a bright room [33]. A petri dish filled with food pellets was fixed at the center of the open field floor. The testing animal was weighed again, then placed in a corner of the apparatus facing the wall and allowed to move freely. The latency from mouse entry to its first bite of food was recorded with a maximum of 10 minutes. The mouse was then returned to its home cage with food supply and the food-taking latency was recorded.

### Multivariate data analysis of the behavior omics

We used multivariate data analysis to gain a comprehensive understanding of all the behavior tests adopted in current study. The behavior omics data included BMS, BMS subscore, alteration percentage (Y-maze), arm return percentage (Y-maze), total arm entry (Y-maze), total distance (Y-maze), novelty preference (NOR), social preference (SI), social novelty preference (SI), latency in home cage (NSF), and latency in novel open field (NSF). The partial least squares discriminant Analysis (PLS-DA) was conducted to classify mice behaviors based on the group effect of age or injury using self-coded R language in RStudio based on the ropls and mixOmics (version 6.20.0) packages [52]. Moreover, the Mantel test was performed to reveal the relationship between mice locomotion and cognition behaviors. The related figure was made through the linkET (version 0.03) R package [18].

### Tissue processing and histopathology

At 60 days post-injury, mice were intracardiac perfused with 4°C saline followed by 4% paraformaldehyde. 10-mm long spinal cord segments centered at the lesion area were dissected out, embedded, and cut into 20-μm-thick sections placed serially on a set of precleaned microscope slides. Luxol fast blue (LFB, Cat# S3382, Sigma-Aldrich) staining was performed to visualize the spared white matter (SWM) as previously [30]. The section of the least amount of SWM was defined as the lesion epicenter. 200 μm and 400 μm rostral and caudal sections to the lesion epicenter were imaged at ×2.5 magnification. The total LFB-positive area of each section was calculated for assessment of SWM by ImageJ software (RRID:SCR_003070). For assessment of lesion volume, 5 mm rostral to 5 mm caudal sections spaced 1 mm apart around the lesion epicenter were stained with GFAP (1:1000; Cat# Z0334, Dako) and DAB (Cat# PK-6100, Vector Labs), then imaged in the same way. Quantification was conducted through the Cavalieri method in Stereo Investigator Software (MBF Biosciences) as previously described [31, 32].

### NanoString transcriptomic analysis

Following intracardiac perfusion with ice cold normal saline, the mouse brain was harvested. Bilateral somatosensory cortex and hippocampus were dissected out and fast-frozen in dry ice. Total RNA was extracted using RNeasy mini kit (Qiagen) with concentration measured by and sent to the Institute for Genome Sciences at University of Maryland School of Medicine for RNA quality test and run for NanoString Neuropathology panel. The transcriptomic data was grouped based on the factors of age plus injury and normalized in NanoString nSolver software (version 4.0). Differential expression analysis was performed based on injury or age. Further in-depth analyses were carried out with self-coded R language in RStudio. Heatmaps were generated based on the ComplexHeatmap package (version 2.13.2) [15]. Volcano graphs were created via the EnhancedVolcano package (version 1.11.3) [5].

### Flow cytometry

Bilateral hemispheres of mice brains were isolated after sacrifice as described above. The sample was mechanically ground into mince and passed through a 70 μm filter in complete Roswell Park Memorial Institute (RPMI) 1640 (Cat# 22400105, Invitrogen) medium. Then enzymic digestion was carried out by collagenase (Cat# 10269638001, 1 mg/mL; Roche Diagnostics), papain (Cat# LS003119, 5 U/mL; Worthington Biochemical), DNAse I (Cat# 10104159001, 10 mg/mL; Roche Diagnostics) with 0.5 M EDTA (Cat# 15575020, 1:1000; Invitrogen) at 37 °C on a shaking incubator (200 rpm) for 1 hour. The cell suspension was processed and transferred into FACS tubes, then incubated with Fc Block (Cat# 101320, Clone: 93; Biolegend) for 10 min on ice and stained for the following surface antigens: CD45-eF450 (Cat# 48-0451-82, Clone: 30-F11; eBioscience), CD11b-APC/Fire^™^750 (Cat# 101262, Clone: M1/70; Biolegend), and Ly6C-APC (Cat# 128016, Clone: HK1.4, Biolegend). Cells were then washed in FACS buffer, fixed in 2% paraformaldehyde for 10 min, and washed once more prior to adding 500 uL FACS buffer. Intracellular staining for Lamp1-PerCPCy5.5 (Cat# 121626, Clone: 1D4B; Biolegend), Thy1-AF700 (Cat# 105320, Clone: 30-H12; Biolegend), NeuN-PE (Cat# FCMAB317PE, Clone: A60; END Millipore), CD68-PE/Cy7 (Cat# 137016, Clone: FA-11; Biolegend), Myelin CNPase-AF647 (Cat# 836408, Clone: SMI91; Biolegend), Synaptophysin-AF488 (Cat# MAB5258A4, Clone: Sy38; END Millipore), ATG7-AF700 (Cat# FAB6608N, Clone: 683906; R&D Systems) and P62/sqstm1-AF647 (Cat# 42822AF647, Novus Biologicals) was performed after fixation/permeabilization. Cyto-ID Autophagy Detection Kit (Cat# ENZ-51031-K200, Enzo Life Sciences) was used according the manufacturer’s instructions.

Data were acquired on a BD LSRFortessa cytometer using FACSDiva 6.0 (BD Biosciences) and analyzed using FlowJo (Treestar Inc.). Countbright^™^ Absolute Counting Beads (Invitrogen) were used to estimate cell counts per the manufacturer’s instructions. Data were expressed as either cells/mg tissue weight. Leukocytes were first gated using a splenocyte reference (SSC-A vs FSC-A). Singlets were gated (FSC-H vs FSC-W), and live cells were gated based on Zombie Aqua exclusion (SSC-A vs Zombie Aqua-Bv510) [49, 50].

### Blood collection and EVs isolation

Blood was collected into precoated EDTA tubes (Cat# 365974, BD Biosciences) through terminal cardiac puncture from each mouse under anesthesia. The blood samples were immediately centrifuged at 500g for 15 min, 2500g for 10 min, and 2500g for 10 min to generate platelet free plasma (PFP), which was aliquoted into multiple tubes and stored at −80°C for later analysis or other applications. Total plasma EVs were isolated by centrifuging at 100,000g for 90 minutes at 4°C with supernatant removed as previously described [22]. For tissue EVs isolation, about 10 mm mouse spinal cord around the lesion area was dissected after perfusing with ice-cold saline. The tissue samples were digested by collagenase (Cat# LS004176, 40U/ml, Worthington Biochemical), then mixed with protease inhibitor (Cat# 11697498001, Millipore Sigma) and phosphatase inhibitor (Cat# 4906837001, Millipore Sigma), next centrifuged at 300g for 5 minutes, 2000g for 10 minutes, 10,000g for 30 minutes, and 100,000g for 70 minutes at 4 °C with the supernatant from each step. The final supernatant was removed carefully, and the remaining tissue EVs pellet was resuspended in 50 μl of PBS. Detailed protocols including Nanoparticle Tracking Analysis (NTA) can be referred to our previous study [22].

### EVs Western Blot

Equal volumes of resuspended plasma EVs sample pellets were loaded onto 4–15% Criterion^™^ TGX Stain-Free^™^ Precast gels (Cat# 5678083, Bio-Rad) and transferred onto nitrocellulose membranes (Cat# 1704159, Bio-Rad). Membranes went through blocking with 5% nonfat milk in PBS plus 0.1% Tween 20 (PBS-T) for 1 hour at room temperature (RT), incubation with primary antibodies overnight at 4°C, washing three times with PBS-T, incubation in species-specific, horseradish peroxidase (HRP)-conjugated secondary antibodies for 1 hour at RT, washing three times with PBS-T prior to the addition of chemiluminescence substrate (Cat# 37071, Thermo Fisher Scientific). The Western Blot image was visualized in the ChemiDoc^™^ MP Imaging System (Bio-Rad), and protein bands were analyzed by Image Lab^™^ software Version 6.0.1 (Bio-Rad). The following antibodies were used: anti-CD63 (Cat# PA5–92370, 1:1000; Thermofisher Scientific) and HRP-conjugated goat anti-Rabbit (Cat# 111-035-003, 1:3000; Jackson ImmunoResearch Laboratories).

### EVs cargo by FirePlex^®^ microRNA assay and analysis

The total plasma and tissue EVs of all experimental groups were used for miR assay including 65 microRNAs through FirePlex^®^ technology (Abcam) which detected microRNAs and measured their abundance by fluorescence intensity. The data was analyzed and visualized as previously described [22].

### Primary mouse glial cell culture, EVs stimulation, and cytokines ELISA assay

Neonatal C57BL/6 mouse cerebral cortex was used to culture microglia and astrocytes as previously described [31, 54]. Primary mixed glial cells were grown in Dulbecco’s Modified Eagle’s Medium/F12 supplemented with 10% fetal bovine serum (FBS) and 1% Pen/Strep (complete media) under 5% CO2 at 37°C. After 10–14 days of incubation, microglia or astrocytes were plated in 96-well plates and incubated with the complete media for 24 h or 2 d, correspondingly. The media was then replaced by adding 2% FBS containing media for serum starvation, which lasted for 1 h for the microglia or 24 h for the astrocytes, followed by 24 h incubation with 2 – 6 × 10^9^ EVs/ml from Young SCI or Sham groups, or 4 × 10^9^ EVs/ml from Aged SCI or Sham groups, or PBS. The resulting supernatants were subjected to CXCL2/MIP-2 (Cat# MM200, R&D Systems) or IL-6 ELISA (Cat# M6000B, R&D Systems) according to the manufacturer’s instructions.

### Primary mouse neuronal culture and Western Blot

Mouse cerebral cortex was dissected from 16–19 days embryo as described previously [31, 54]. Dissociated cells were seeded in 12-well plates pre-coated with poly-D-lysine (50 μg/ml, 70–150 kDa, Cat# P6407, Sigma-Aldrich) and cultured with Neurobasal^™^ media (Cat# 21103049, Gibco) supplemented with 2% B-27 (Cat# 17504044, Gibco), 0.5mM Glutamax (Cat# 35050061, Gibco), and 2% penicillin-steptomycin (Cat# 15140122, Gibco) for 7 days. After 24 h incubation with 6 × 10^9^ EVs/ml from Young SCI or Sham groups, 4 × 10^9^ EVs/ml from Aged SCI or Sham groups, the neurons were harvested and processed with RIPA lysis buffer (Cat# R0278, Sigma-Aldrich) supplemented with 1× Protease Inhibitor Cocktail, Phosphatase Inhibitor Cocktail II & III (Cat# P8340, P5726, P0044, Sigma-Aldrich) for protein determination using the Pierce BCA Assay Kit (Cat# 23225, Thermo Fisher). Western Blotting was performed as described above. The following primary antibodies were used: cleaved caspase 3 (Cat# 9661S, 1:500, Cell Signaling Technology), actin/β-actin (Cat# A1978, 1:10,000, Sigma-Aldrich).

### Statistical analysis

All data are presented as mean ± SEM from the indicated number of independent experiments. All behavioral, histological, and ex vivo studies were performed by investigators blinded to group designations. Statistical analysis was performed using GraphPad Prism 8.4.2 (GraphPad Software, LLC) for most bar graphs, or SigmaPlot 12.0 (Jandel Scientific, San Jose, CA, USA) for BMS score and subscore, or R for transcriptomic data. Normal distribution of data was assessed with the Shapiro-Wilk test. For multiple comparisons, one-way or two-way ANOVA were performed followed by Tukey’s multiple comparisons post-hoc test for parametric (normality and equal variance passed) data. Nonparametric data were analyzed by Mann-Whitney test. For analysis of miR assay data, two-way ANOVA test was used and followed by Tukey’s multiple comparisons test to compare main effects of age or injury. BMS scores and subscores were analyzed with two-way ANOVA for repeated measurements followed by Sidak’s multiple comparisons post-hoc test. Significance was set at p ≤ 0.05 and detailed in figure legends.

## Results

### Old age exacerbates motor functional deficits and neurological dysfunction after SCI

To determine whether old age impacts neurobehavioral outcomes after SCI, we assessed young adult and aged male C57BL/6 mice using a battery of behavioral tests. Body weight of animals was monitored before and after injury (Fig. S1a). Our data showed that the body weights of 18-month-old mice were significantly higher than those of 10–12 weeks old mice (2.5–3.0 months old). However, SCI-induced weight loss was evident at 49d post-injury in both young and the older group. Actual injury force and displacement were detailed in Fig. S1b. Although no significant differences between groups in the actual force were observed, the resulting displacements of the heavier aged mice were significantly lower than those generated from young animals (Unpaired t test, *p<0.05), suggesting that total body weight might affect displacement of the cord during injury.

**Fig. 1:**
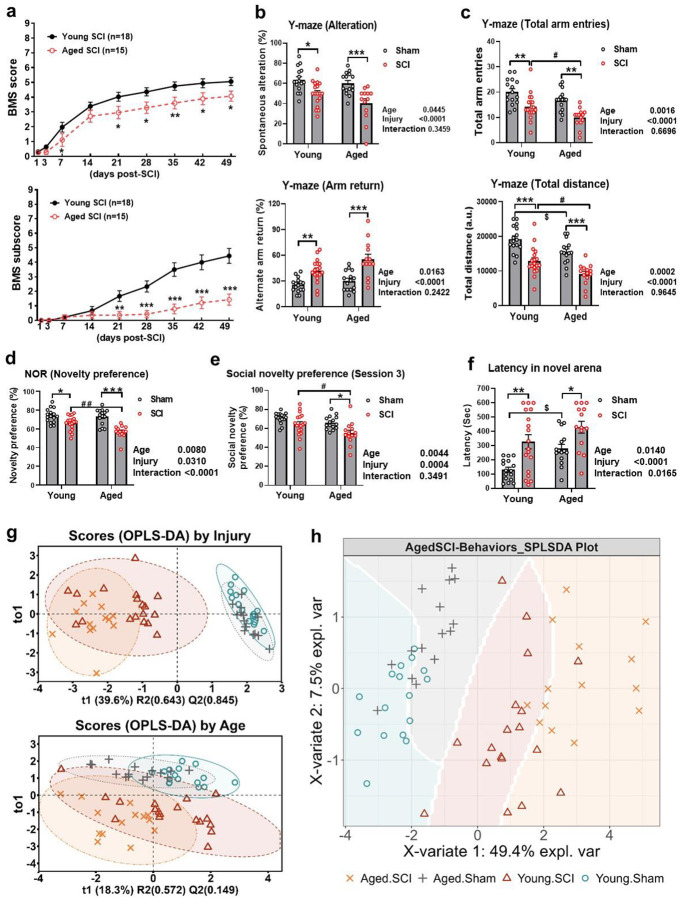
Aged mice exhibit exacerbated motor functional deficits and neurological dysfunction at 2 months after SCI**. a** Locomotor function was measured by BMS score and BMS subscore in both young adult and aged mice at multiple post-injury time points. N=18 (Young SCI) and 15 (Aged SCI). Two-way ANOVA with repeated measurement was performed combined with Young Sham (n=16) and Aged Sham (n=15). **b** Cognition function was assessed in Y-maze test as alteration and arm return. **c** Motor function was assessed in Y-maze test as total arm entries and total distance. Novelty preference in novel object recognition (NOR) test (**d**), social novelty preference in social recognition (SR) test (**e**), and latency in novel arena in novelty suppressed feeding (NSF) test (**f**) were calculated and compared among the four groups. **g** OPLS-DA of the behavioral data was performed by injury or age. **h** Integrated behavioral data was applied for SPLSDA as clusters by group. Two-way ANOVA with Tukey’s post hoc test was performed for (**b-f**). *, #, $: *p* < 0.05; **, ##: *p* < 0.01; ***: *p* < 0.001.

Longitudinal hind limb motor function was evaluated using the BMS on day 1 and day 3 after injury and weekly thereafter for up to 7 weeks (Fig. 1a). Both aged and young sham mice had full scores of 9. After moderate SCI, all injured mice showed rapid increase of BMS score in the first two weeks, indicating a spontaneous recovery, and reached a plateau after 4 weeks post-injury. By day 7 after injury, aged mice had significantly reduced BMS scores compared with young animals. Significant differences between groups remained through 49 d after injury. Beginning 21 d post-injury, BMS sub-scores of Aged SCI group were significantly lower than Young SCI group. This reduction remained through 49 d after injury. Together these data show that old age worsens locomotor functional deficits after SCI.

Cognitive function was measured using the Y-maze for hippocampus-dependent spatial working memory and novel object recognition (NOR) test for recognition memory. In the Y-maze test, the percentage of spontaneous alteration and alternate arm return were adopted to access the short-term memory of the four groups. Aged mice had comparable baseline level of alternation but higher one of arm return compared to young mice before SCI (Fig. S1c). After the injury, both aged and young mice showed significantly lower spontaneous alteration and higher arm return versus sham animals (Fig. 1b). On the other hand, aged mice showed significantly lower total arm entries and total distance compared to young mice in the baseline (Fig. S1d) and post-injury phase (Fig. 1c). Statistical analysis indicated significant group effects of age and injury for these parameters, which suggested that Aged SCI group had more severe working memory impairments and decreased locomotion activities. In the NOR test, all mice took comparable time exploring the two identical objects in the open field during sample phase, indicating no object bias (Fig. S1e). In choice phase, however, the time for exploring the newly-introduced object of Aged SCI group was further reduced versus Young SCI group, resulting in significantly lower novelty preference (Fig. 1d). Statistical analysis indicated significant group effects of age and injury as well as their interaction, which implied that learning and memory in aged mice was further impaired after SCI compared to the young group.

To assess depression-like phenotype, we subjected mice to three-chamber social recognition (SR) test and novelty suppressed feeding (NSF) test. With an unbiased preference to the chambers (Fig. S1f), the four groups showed comparable social preference to a stranger mouse than an empty cup (Fig. S1g). When another new mouse was introduced, only Aged SCI group showed significantly lower social novelty preference compared to other groups (Fig. 1e). The results suggest potential functional deficits in olfaction, memory or social interest mediated by SCI in aged mice. In NSF test, the four groups showed no difference of food-taking latency in home cages with significant age effect (Fig. S1h). While in the novel arena, significant group effects of age and injury and their interaction were observed based on the difference between sham groups and the increases of food-taking latencies after SCI (Fig. 1f). These data suggest that the aged mice are more vulnerable to stress, anxiety, and depression after SCI.

To reveal the relationship between motor functional deficits and cognitive impairments following SCI, we used Mantel Test to estimate the correlations between the two behavioral sets, locomotion and cognition (Fig. S1i). The results suggest that locomotion has significant correlations with memory-related behaviors but not with depression-like behaviors, and cognition has significant correlations with most other behaviors. There are barely correlations between behaviors in NSF and others. To plot an overall behavioral profile of different groups highlighting the effects of age and injury, we applied multivariate analysis to all the behavioral data in Fig.1a–f through multiple Partial Least Squares (PLS) methods. By defining SCI and Sham as discrete variables for injury effects or Aged and Young for age effects in combination with all the behavioral data, we acquired the scores of each sample in a correlated axis (t1) and its orthogonal uncorrelated axis (to1). As Fig. 1g showed, two injury groups and two sham groups cluster together into separated pools along the axis that had a 39.6% loading of injury effects. While two aged groups and two young groups overlap heavily along the axis with a 18.3% loading of age effects. These results imply that injury caused significant shifting of behavioral profile while aging had more complicated impacts in the current study. Moreover, all the behavioral data were applied for Sparse Partial Least Squares Discriminant Analysis (SPLS-DA) based on their group information. Fig. 1h displayed the mapping plot for the first two components. The four groups showed distinct profiles from each other, suggesting that the fundamental differences in age and injury are highly correlated with their behaviors of locomotion and cognition.

### Aged mice exhibit larger tissue damage following SCI

**Fig. 2:**
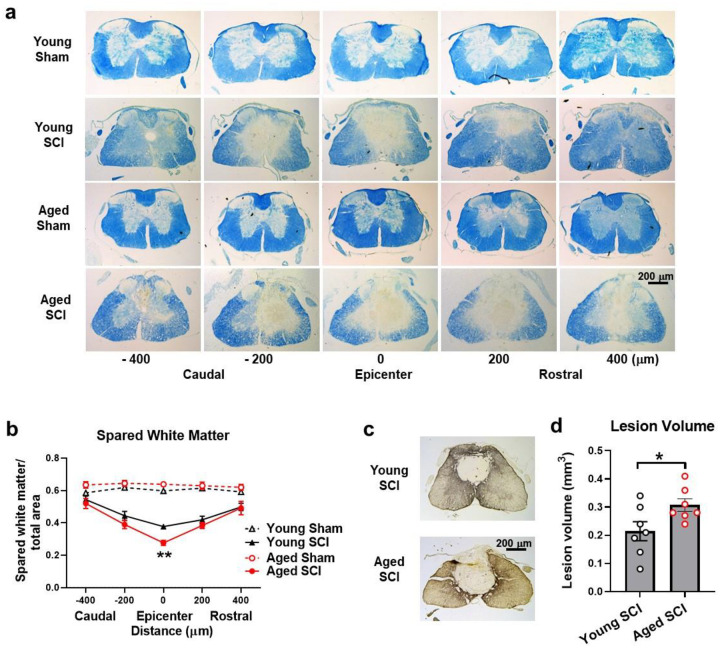
Old age exacerbates tissue damage at 2 months after SCI. **a** Representative Luxol fast blue (LFB) stained sections at 2 mm rostral or caudal to the epicenter of each subject illustrate the differences in myelinated spared white matter (SWM) area between young and aged animals. **b** SWM area was quantified at 2-mm intervals rostral and caudal to the injury epicenter. Unpaired t test was performed for two injured groups at different sites. **c** Representative GFAP-DAB staining images of spinal cord sections showed the lesion area at the epicenter of young or aged mouse. **d** The average lesion volume was assessed. Unpaired t-test was performed. Scale bar=200 μm. n=7 mice/group, *p < 0.05; **p < 0.01.

To determine if the observed behavioral exacerbation in aged mice may relate to decreased remyelination of spared axons, spinal cord sections from injured mice perfused at 8 weeks were stained with Luxol fast blue (LFB) and spared white matter (SWM) area was quantified at 2-mm intervals rostral and caudal to the injury epicenter. Representative LFB stained sections at 2 mm rostral or caudal to the epicenter of each subject illustrate the differences in myelinated WM area between young and aged animals (Fig. 2a). Statistical analysis indicated that aged mice had significantly reduced SWM at the epicenter after SCI compared to the young animals (Fig. 2b). Furthermore, SCI-induced lesion volume/cavity formation was measured with GFAP/DAB staining at 8 weeks after SCI and analyzed by unbiased stereological techniques (Fig. 2c,d). The average lesion volume assessed for the aged mice was significantly enlarged compared to young animals. This expansion occurred in both white and gray matter, with an overall increase in cavity formation and tissue loss. Together, these histopathology data show a positive association between SWM, lesion volume, and locomotor functional deficits after chronic SCI, which is exacerbated in older mice.

### SCI in young and aged mice lead to distinct RNA transcriptome profiles in the brain

**Fig. 3:**
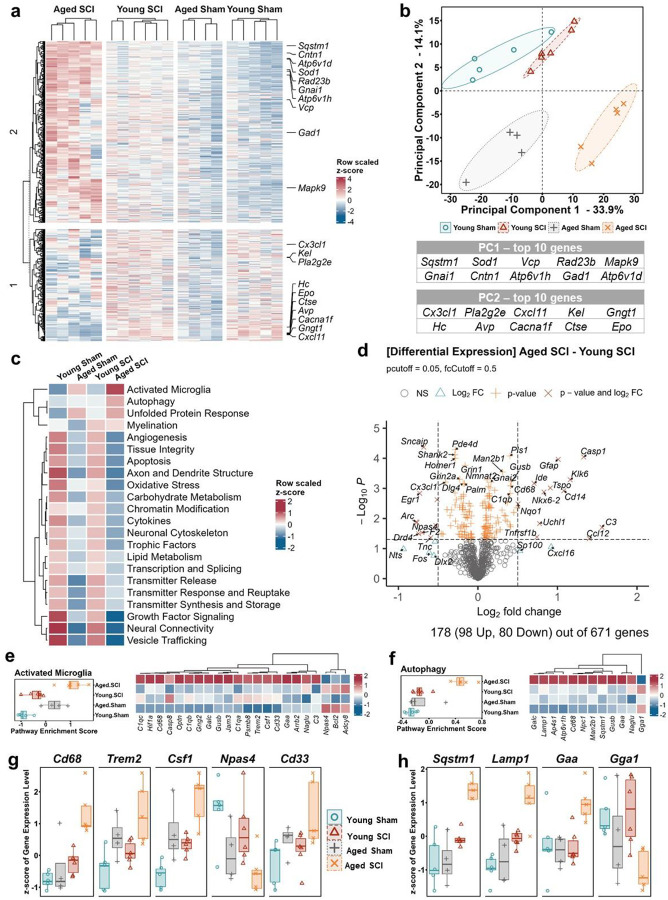
SCI leads to distinct RNA transcriptome profiles in the somatosensory cortex between aged mice and young adult animals. **a** The genes in mouse cortex exhibited different expression profiles by group in the heatmap labeled with top 10 genes from the 1^st^ and 2^nd^ principal components in PCA analysis. **b** The four groups clustered into separated pools in PC1 × PC2 dimensions based on the PCA analysis of mouse cortical transcriptomic data. **c** Pathway scoring showed up/down-regulation of gene expression enriched in different pathways of Aged SCI mice compared to other groups. **d** Gene differential expression analysis between Aged SCI and Young SCI was visualized as volcano graph. **e-h** Pathway enrichment analysis indicated specifically upregulations of activated microglia and autophagy in Aged SCI mice, showing typical genes with expression variations relating to inflammation (**g**) and autophagy (**h**). n=4–6 mice/group.

To address age-related differences in the transcriptional response to SCI, somatosensory cortical and hippocampal tissues were sampled from young and old mice at 2 months post-injury. Using the NanoString Neuropathology panel, we examined transcriptional changes for 770 genes within six fundamental themes of neurodegeneration: neurotransmission, neuron-glia interaction, neuroplasticity, cell structure integrity, neuroinflammation, and metabolism. The mRNA reading counts of all the genes in the cortex was scaled by individual mouse and clustered into two blocks by K-means method based on Aged SCI group (Fig. 3a), in which block 1 contained downregulated genes while block 2 included upregulated ones. Principal Components Analysis (PCA) of the same datasets demonstrated the in-group similarities as well as between-group differences (Fig. 3b). PC1 was the major principal component with a contribution of 33.9% gene variations separating injury groups from sham groups, which may represent injury effects. PC2 contained 14.1% gene variations and separated aged mice from young groups, which may represent age effects. The top 10 genes with most contributions in PC1 or PC2 assembled correspondingly in block 2 or block 1 of the heatmap (Fig. 3a), suggesting that SCI plays important roles in the upregulations of the genes in block 1 and aging contributes to the downregulations of block 2 genes in the cortex of Aged SCI mice.

Through heatmap hierarchy clustering of the average z-scores for each group, we identified “Activated Microglia” and “Autophagy” as the top two upregulated pathways in the cortex between young and aged SCI mice (Fig. 3c). Table S1 listed the two-way ANOVA statistical analysis results followed by age, injury, and their interaction. To reveal the gene variations relating to these pathways, we performed differential expression (DE) analysis between Aged SCI and Young SCI groups, which showed that 178 out of 671 genes changed significantly at mRNA level (Fig. 3d). Furthermore, transcriptomic changes of these genes involved in the two pathways were displayed as z-score heatmaps of the four groups, showing robust differences of Aged SCI group compared to others (Fig. 3e, f). Based on the statistical analysis of relevant gene sets (Table S2), mRNA levels of typical individual genes were displayed in Fig. 3g for Activated Microglia pathway, and in Fig. 3h for Autophagy pathway. Aged SCI mice exhibited high levels of *Cd68*, *Trem2*, *Csf1*, and *Cd33* with significant age and injury group effects (Table S2), indicating upregulated activities of microglia and myeloid and lymphocytes infiltration (Fig. 3g). Reduction of the neuroprotective transcription factor *Npas4* gene was also observed in the brain from aged SCI mice (Fig. 3g). *Sqstm1*, a key gene for autophagosome formation, along with *Lamp1* and *Gaa* that reflect lysosome activities, were highly upregulated with significant group effects of age and injury (Fig. 3h, Table S2). However, Gga1, a gene mediating cargo transport from the trans-Golgi network to endosomes and lysosomes, was downregulated (Fig. 3h). These results suggest that autophagy processes were dysregulated in the aged cortex following SCI.

**Fig. 4:**
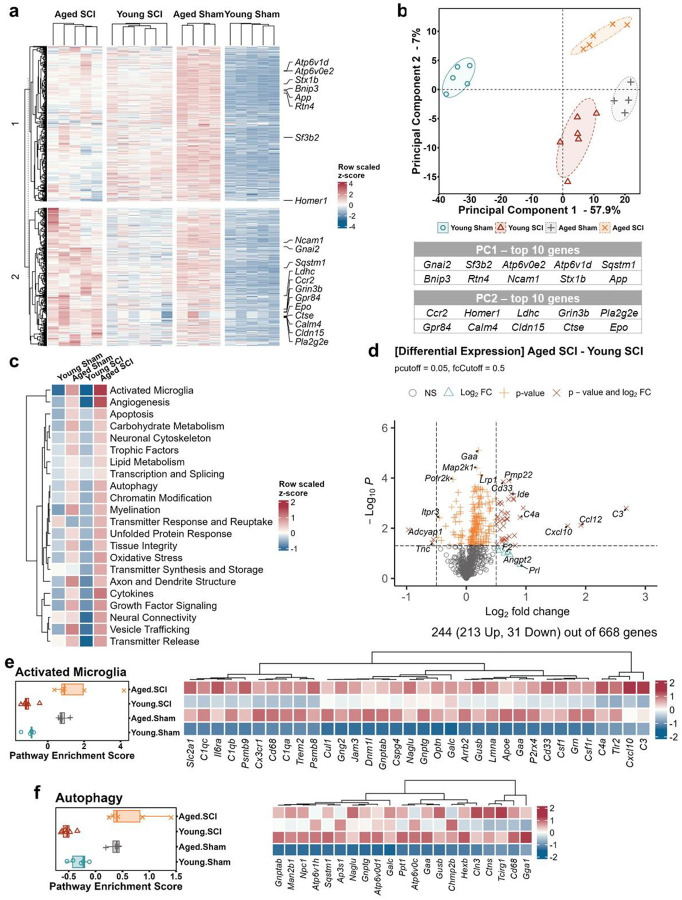
Old age and SCI lead to distinct RNA transcriptome profiles in mouse hippocampus. **a-b** The heatmap of gene expression z-scores in mouse hippocampus formed different clusters by group labeled with top 10 genes from PCA analysis (**b**). **c** Pathway scoring showed major aging effect in the upregulations of most pathways in aged groups. **d** Gene differential expression analysis between Aged SCI and Young SCI was visualized as volcano graph. Pathway enrichment analysis of activated microglia (**e**) and autophagy (**f**) indicated that gene variations relating to inflammation and autophagy were upregulated by aging with SCI. n=4–6 mice/group.

To further analyze the connection between aging, SCI, and the behaviors reflective of cognitive impairment and depression that we observed in tested mice, hippocampal tissue was also analyzed. In the hippocampus, the identical genes of the neuropathology panel were clustered into two blocks through the four groups (Fig. 4a). Compared to the baseline in Young Sham mice, most genes were universally upregulated in aged groups and young injured mice, exhibiting distinct profiles from each other. PCA analysis showed the transcriptomic profiles of individual animals clustered into well-separated group pools (Fig. 4b). Most of the pathways were upregulated in aged hippocampus compared to young mice (Fig. 4c). Based on the DE data (Fig. 4d), we investigated the pathways of Activated Microglia and Autophagy (Fig. 4e, f). Two-way ANOVA analyses demonstrated that there are significant effects of aging but neither injury nor their interaction among the four groups of the two pathways (Table S3). The statistical analysis results of the relevant gene sets were included in Table S3.

Taken together, our results show that SCI leads to distinct transcriptomic profiles and diverse pathway regulations in old age and young adult mice as well as in different brain regions. Upregulation of activated microglia and dysregulation of autophagy-lysosome pathway in aged groups following SCI are pronounced.

### Old age increases infiltration of lymphocytes and exaggerates microglial responses to SCI

**Fig. 5:**
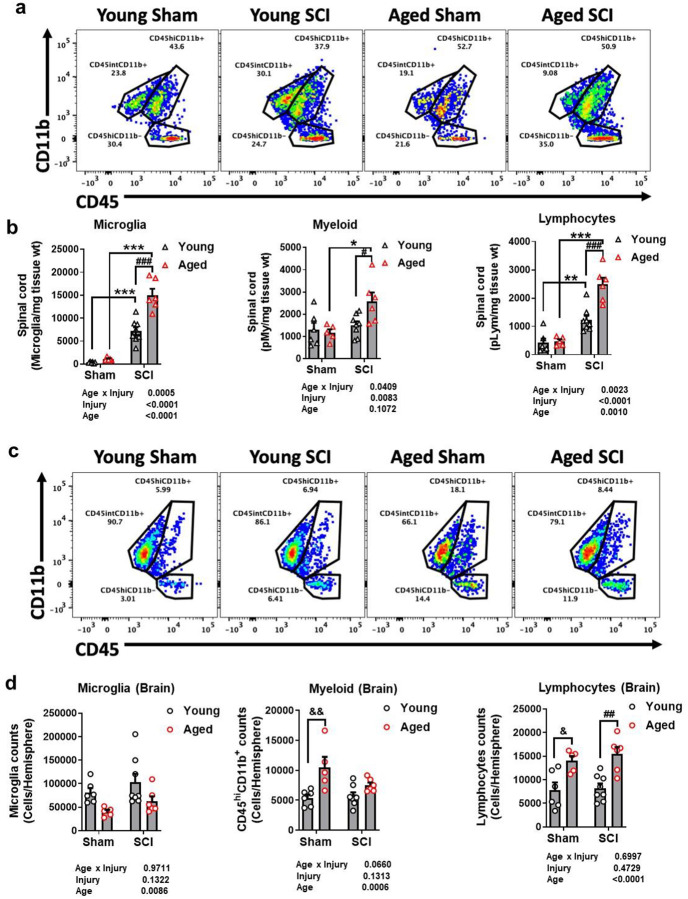
Old age increases infiltration of lymphocytes and exaggerates microglial responses to SCI. Young and aged C57BL/6 mice were subjected to moderate SCI and both lesion area and the brain were extracted at 2 months post-injury for flow cytometry. **a-b** Representative dot plots depict the relative immune cell composition in the lesion area of the spinal cord at 2 months (mon) post-injury (**a**). Cell counts normalized by tissue weight showed increased number of CD45^int^CD11b^+^ microglia, CD45^hi^CD11b^+^ infiltrating myeloid cells, and CD45^hi^CD11b^−^ infiltrating lymphocytes in the spinal cord of SCI mice (**b**). **c-d** Representative dot plots depict the relative immune cell composition in the brain (**c**). Decreased microglia along with increased myeloid and lymphocytes in aged brain showed significant group effect of aging rather than injury or their interaction compared to the young groups (**d**). Data was represented as mean ± SEM for Aged SCI group (n=6), Young SCI group (n=8), Aged Sham group (n=5), and Young Sham group (n=6). Two-way ANOVA with Tukey’s post hoc test was performed for (b, d). *, #, &: *p* < 0.05; **, ##, &&: *p* < 0.01; ***, ###: *p* < 0.001.

Next, we investigated the cellular inflammatory response at 2 months after SCI. Microglia and leukocyte identification and characterization in both lesion area and the brain were performed using flow cytometry. The expression levels of CD45 and CD11b were used to distinguish microglia (CD45^int^CD11b+), myeloid (CD45^hi^CD11b+), and lymphocyte (CD45^hi^CD11b-). At the injury site (Fig. 5a), SCI induced significantly increased number of microglia and lymphocytes in both young and aged groups. Greater myeloid infiltration was observed in aged SCI mice compared to aged sham animals, but not in young injury mice at this time-point. Nevertheless, marked microglial accumulation and increased infiltration of myeloid, as well as putative lymphocyte populations, were found in old mice at 2 months post-injury. Statistical data showed that the groups effects of age, injury, and their interaction are significant (Fig. 5b).

In the brain, decreased numbers of microglia were found in aged compared to young adult mice (Fig. 5c). Statistical analysis showed an effect of age, but not injury. This is in agreement with the reports previously by our group [50] and others [69] and may reflect age-related dystrophy and proliferative senescence. Although aged SCI mice showed further elevation of lymphocyte accumulation compared to young SCI mice, increased infiltration, and accumulation of myeloid and lymphocytes were detected in aged brain with significant age effect only (Fig. 5d).

### Functions of microglia and neurons in the brain after SCI are altered with age

**Fig. 6:**
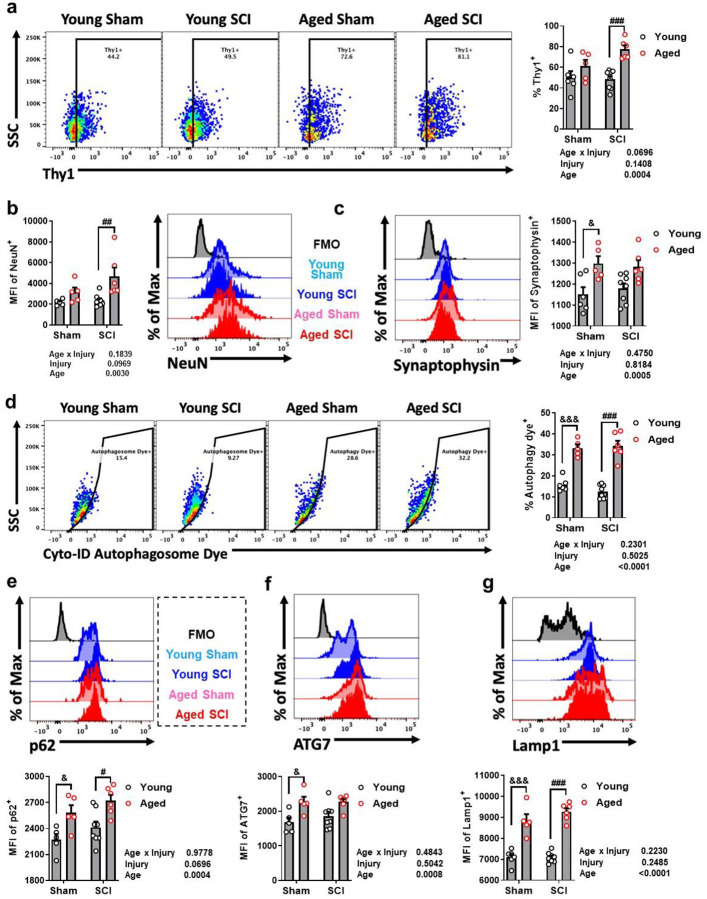
SCI in aged mice alters microglia function and dysregulates microglia autophagy in the brain. **a** Representative dot plots and percentage of Thy+ cells in the microglia. **b-c** Representative histograms and MFI of NeuN (**b**) and Synaptophysin (**c**) in the microglia. **d** Representative dot plots and percentage of Cyto-ID Autophagosome Dye+ cells in the microglia. **e-g** Representative histograms and MFI of p62 (**e**), ATG7 (**f**), and Lamp1 (**g**) in the microglia. Data was represented as mean ± SEM for Aged SCI group (n=6), Young SCI group (n=8), Aged Sham group (n=5), and Young Sham group (n=6). Two-way ANOVA with Tukey’s post hoc test was performed for (a-g). #, &: *p* < 0.05; ##: *p* < 0.01; ###, &&&: *p* < 0.001. SSC: side scatter; FMO: fluorescence minus one; MFI: Mean fluorescence intensity.

To better understand the effects of age on microglia function in the brain following SCI, flow cytometry was performed to examine phagocytosis and autophagy which were informed largely by our NanoString results indicated age-related, posttraumatic changes in these pathways after injury. Microglial phagocytosis was measured by intracellular detection of neuronal antigens, including Thy1, NeuN, and Synaptophysin. Consistent with our previous reports [49, 50], microglial cells in aged groups showed higher levels of these neuronal markers with significant group effect of age, confirming increased activities of microglial phagocytosis in aged brain (Fig. 6a–c). Although the group effect of injury was not significant, the two-way ANOVA multiple comparisons tests indicated significant increases of Thy1 and NeuN for Aged SCI group versus Young SCI group (Fig. 6a, b) as well as Synaptophysin for Aged Sham versus Young Sham (Fig. 6c). These findings suggest that microglial phagocytosis of dead or dying neurons increase in brain with both age and SCI, consistent with our gene expression data.

We recently reported that age-related deficits in autophagy function underlie chronic microglial activation and dysfunction following brain trauma [50]. Therefore, we examined lysosome and autophagosome content in microglia (Fig. 6d–g). Aged mice showed greater formation of LC3-positive autophagosomes and increased expression level of p62 (*Sqstm1*), ATG7, and Lamp1, compared to young mice. Moreover, the two-way ANOVA multiple comparisons tests showed that several lysosomes and autophagosome components in microglia from aged SCI mice were significantly increased compared to young SCI animals. These data indicate that brain microglial phagocytosis and autophagic function are exacerbated with age and SCI, consistent with our NanoString gene signature.

We have previously shown an approach for identifying neuronal populations in the brain using flow cytometry [48]. Using the same strategy, we examined changes in neuronal function of the brain following SCI. Reduced expression levels of synaptophysin and myelin CNPase were detected in neurons of aged groups with significant group effect of age (Fig. S2a, b). The percentages of neurons positive for CD68, a lysosomal/endosomal membrane marker, were significantly higher in the brains from aged mice versus young animals (Fig. S2c). The two-way ANOVA multiple comparisons tests indicated significant reduction of myelin CNPase and increase of CD68 in Aged SCI mice versus Young SCI group (Fig. S2b, c). Among the markers of lysosome and autophagosome, we did not detect significant difference of LC3-positive autophagosomes or Lamp1 or injury effect in neuronal population among the groups (Fig. S2d-g). However, SCI induced higher ATG7 expression level and lower p62 level in aged mice compared to young adult animals tested in the two-way ANOVA multiple comparisons (Fig. S2e, f).

### Age may exacerbate the plasma extracellular vesicles (EVs) response after SCI

**Fig. 7:**
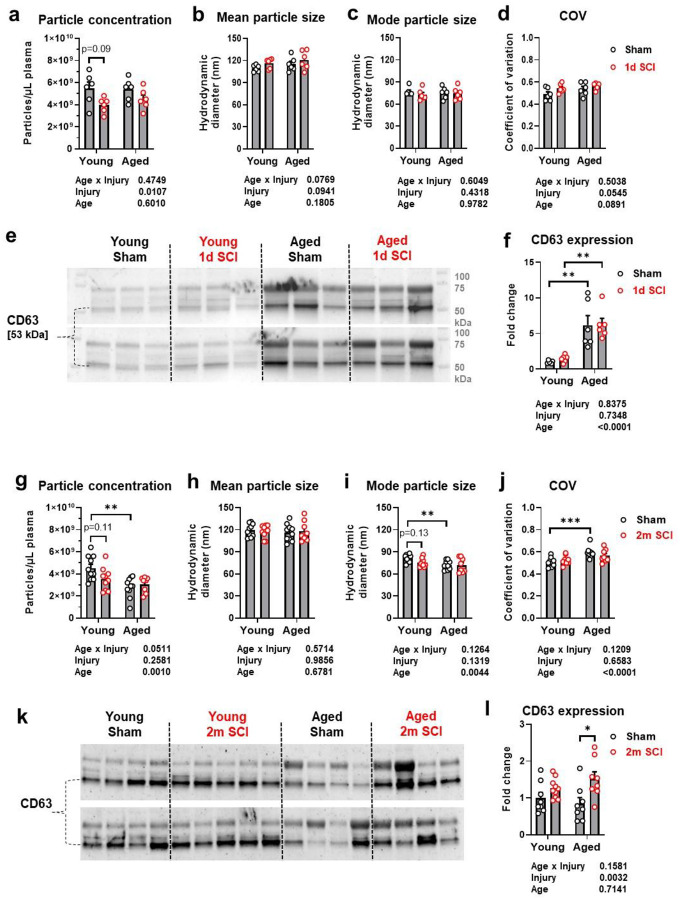
Age may exacerbate the plasma EV response after SCI at 1d and 2 months post-injury. **a-f** EV particle parameters (**a-d**) were analyzed by Nanoparticle Tracking Analysis (NTA) of one day post-injury in aged (18-month-old mice) and young adult (10–12-week-old) animals. Western Blot images showing significantly upregulated CD63 expression in aged mice compared to young groups (**e-f**). g**-l** EV particle parameters (**g-j**) were analyzed by NTA of 2 months post-injury in 21–22-month-old mice and 20-week-old animals. Western Blot images showing significantly upregulated CD63 expression in aged SCI mice compared to young SCI groups (**k-l**). n=6 mice/group (**a-f**) and 8–10 mice/group (**g-l**). Two-way ANOVA with Tukey’s post hoc test was performed. *p < 0.05, **p < 0.01, ***p<0.001.

We have recently evaluated changes in plasma EVs after SCI in young adult mice up to 2 weeks post-injury and showed that plasma EVs at 1d post-injury had a significantly altered miR profile and produced a neuroinflammatory response when injected into the brain [22]. Here, we extended these observations in studies examining acute and more chronic time points after injury in both young and aged mice. As SCI causes persistent neuroinflammation and neurodegeneration in the brain [32, 33], we hypothesized that alterations in EVs-mediated signaling may contribute to the process. To address this hypothesis, we evaluated plasma EVs parameters in young adult and aged mice at 1d and 2 months post-injury (Fig. 7). Nanoparticle Tracking Analysis (NTA) was used to measure plasma EVs particle count and size as described previously [22]. While there were no differential changes in particle concentration, particle size, and coefficient of variation (COV) at baseline between aged (18-month-old mice) and young adult (10–12-week-old) animals (Fig. 7a–d), 21–22-month-old mice had lower particle counts, smaller modal particle size and a higher COV compared to 20-week-old animals (Fig. 7g–j). Injury did not significantly alter these parameters in either group. We then analyzed the plasma EV isolates by western blot for expression of EVs marker CD63. At 24h post-injury, we observed increased CD63 expression levels in aged mice compared to young adult mice (Fig. 7e). Statistical analysis showed two-way ANOVA main effect of age, but not related to injury. Although 21–22-month-old mice showed similar baseline of CD63 protein expression compared to 20-week-old animals, chronic injury in these animals increased CD63 expression (Fig. 7k, i). In contrast, young adult mice subjected to 2-month injury did not alter CD63 expression.

### Old age alters EVs miRs content associated with inflammation and autophagy signaling after SCI

**Fig. 8:**
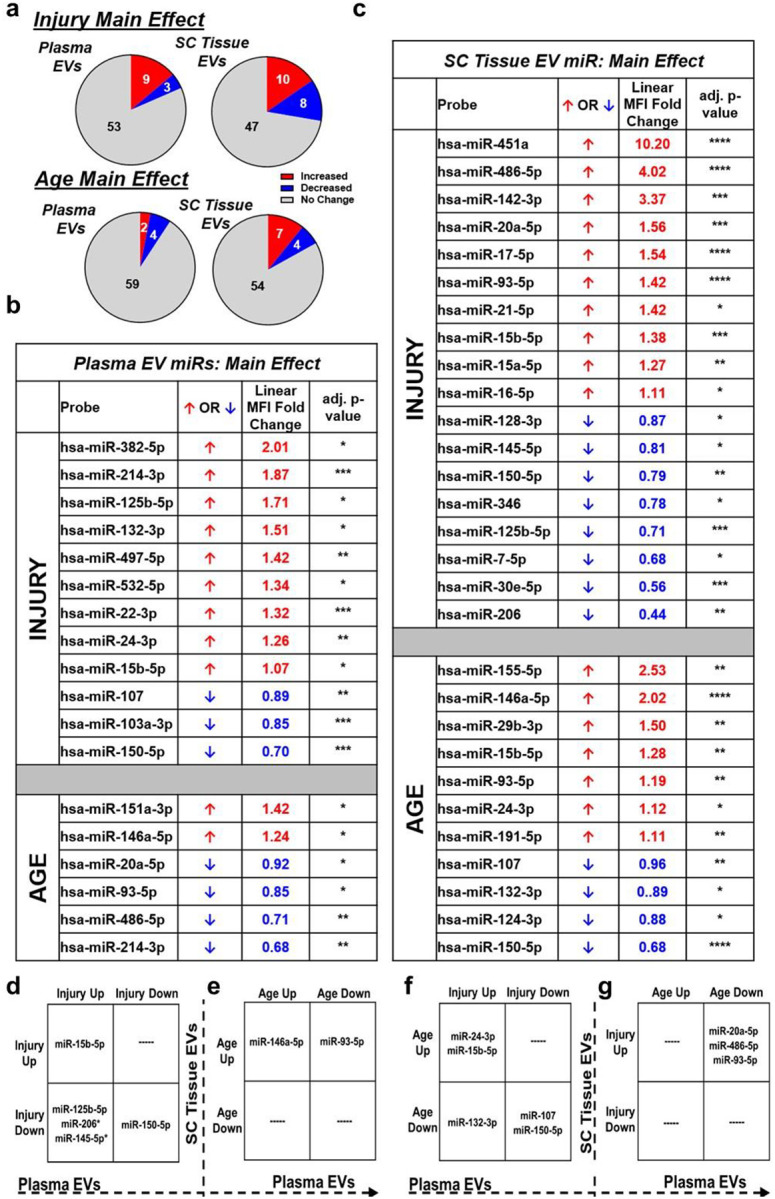
Old age following SCI alters microRNA (miRs) cargo profiles in both plasma and tissue EVs. **a-c** Main effect analysis indicated the numbers of EVs-derived miRs altered by injury or age (a), which were listed as from plasma (**b**) or from spinal cord (SC) tissue (**c**). **d-g** Detailed information showed that different miRs were up/down-regulated in plasma or tissue with age or after injury. n=6 mice/group.

Next, we examined acute changes in EVs cargo in young and aged animals. EVs were isolated from both the plasma and spinal cord (SC) injury site at 1d post-SCI to compare the overall miRs profile using a CNS-enriched Fireplex^®^ assay. Of the 65 miRs tested, main effects analysis revealed 12 miRs (9 up, 3 down) in plasma EVs and 18 miRs (10 up, 8 down) in SC EVs that were differentially expressed after injury as well as 6 miRs (2 up, 4 down) in plasma and 11 miRs (7 up, 4 down) in SC with age (Fig. 8a–c). Corroborating prior reports [9, 16, 44], these age-modified miRs were mainly associated with inflammatory activation (“inflammaging”), including increases in miR-146a-5p and miR-155–5p, and decreases in miR-214–3p, miR-93–5p, and miR-20a-5p.

To address the relationship of miRs changes between the plasma EVs and SC EVs, we compared the data of differentially expressed miRs by injury, age, and the interaction (Fig. 8d–g). The upregulation of miR-15b-5p and downregulation of miR-150–5p were consistent in both samples after injury (Fig. 8d). All three miRs (miR-125b-5p, miR-206, miR-145–5p) decreased after injury in SC EVs and increased in plasma EVs (Fig. 8d). Further analyses indicated that only injury effect was significant for miRs cargo alterations in SC tissue; however, group effects of both age and injury, as well as their interaction, were significant for miRs changes in plasma (Fig. S3a-c.

Another miR (miR-23a-3p) in the plasma EVs and two miRs (let-7d-5p, miR-103a-3p) in the SC EVs were also found to have a significant interaction between the group effects of age and injury (Fig. S3d-f). miR-23a-3p, similar to miR-206 and miR-145–5p, increased in the plasma of young mice following SCI with smaller changes in aged mice (Fig. S3d). In the injured tissue, let-7d-5p increased and miR-103a-3p decreased in Aged SCI mice compared to Young SCI group (Fig. S3e, f).

Moreover, miR-146a-5p, known to modulate neuroinflammation during the progression of Alzheimer’s disease or neuropathic pain [25, 62], increased in both compartments with age in our study (Fig. 8e). miR-93–5p was upregulated in SC EVs with both age and injury but downregulated in plasma EVs with age (Fig. 8e). Two miRs (miR-24–3p and miR-15b-5p) increased in SC EVs with age as well as in plasma EVs after injury, contrasting with miR-107 and miR-150–5p (Fig. 8f). Meanwhile, miR-132-3p was upregulated in plasma after injury and downregulated in SC EVs with age (Fig. 8f). Two miRs (miR-20a-5p and miR-486-5p) were found increased in SC EVs after injury while decreased in plasma EVs with age (Fig. 8g). Among these miRs, several (miR-93–5p, miR-107, and miR-486–5p) were reported to regulate apoptosis and cellular autophagy [26, 28] and some (miR-24–3p, miR-132–3p, and miR-20a-5p) were reported to regulate inflammation response [14, 43]. Collectively, these data show that EVs miRs contents are exacerbated with age and SCI, which are associated with neuroinflammation and disrupted autophagy-lysosome pathways- consistent with our NanoString results and indicating age-related dysregulation in these pathways after injury.

### Circulating EVs from SCI mice induce the secretion of pro-inflammatory cytokines and neuronal apoptosis

**Fig. 9:**
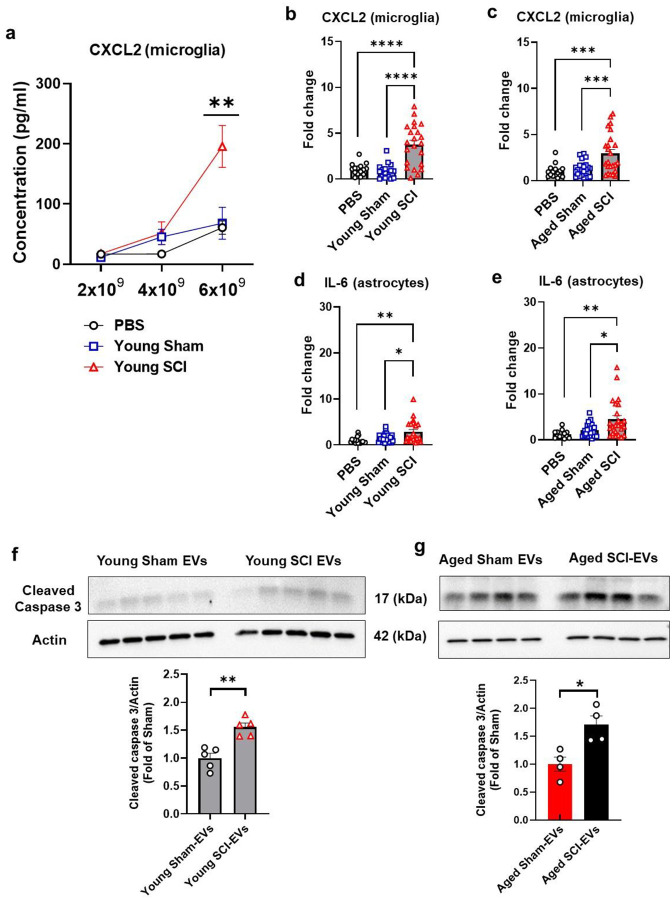
Circulating EVs from SCI mice induce the secretion of pro-inflammatory cytokines and neuronal apoptosis *in vitro*. Primary microglia, astrocytes, and neurons were cultured from mouse neonatal or embryonic cortices. Plasma EVs were isolated from young adult (12 mice) or aged mice (12 mice) at 1 d post-injury or sham mice (12 mice). Nanoparticle Tracking Analysis (NTA) was measured to obtain EVs concentration (EVs particles/ml). **a-b** Primary microglia were exposed to three different dosages (2 × 10^9^, 4 × 10^9^, and 6 × 10^9^ particles/ml) of EVs for 24 h, separately and the resulting supernatants were collected for CXCL2 ELISA assays. Different dosages of Young SCI EVs were tested for the optimal condition with PBS or Young Sham EVs (**a**) as control. The fold changes of CXCL2 levels (**b**) were compared among groups after the stimulation of Young SCI EVs (6 × 10^9^ particles/ml, n=11 mice × 2 independent microglia cultures). Two batches of CXCL2 ELISA assays are presented in Supplemental Figure S4a-c. **c** The fold changes of CXCL2 levels (**c**) were compared among groups after the stimulation of Aged SCI EVs (4 × 10^9^ particles/ml, n=12 mice × 2 independent microglia cultures). Two batches of CXCL2 ELISA assays are presented in Supplemental Figure S4d-f. **d-e** The fold changes of IL-6 levels released from cultured astrocytes were compared among groups after the stimulation of Young SCI EVs (**d**, 6 × 10^9^ particles/ml, n=12 mice × 2 independent astrocytes cultures) or Aged SCI EVs (**e**, 4 × 10^9^ particles/ml, n=12 mice × 2 independent astrocytes cultures) compared to Young Sham EVs (n=12 mice × 2 independent astrocytes cultures) or Aged Sham EVs (n=11 mice × 2 independent astrocytes cultures) or PBS. Two batches of IL-6 ELISA assays are presented in Supplemental Figure S4g-l. *p < 0.05; **p < 0.01; ***p < 0.001; ****p < 0.0001. One-way ANOVA with Tukey’s post hoc test was performed (**a-e**). **f-g** Expression levels of cleaved caspase 3 protein in cultured neurons treated with various plasmas EVs isolated from young adult mice (**f,** 6 × 10^9^ particles/ml) and aged animals (**g,** 4 × 10^9^ particles/ml). Each blot lane represents an individual animal EVs. After 24h treatment, the harvested neuronal samples were blotted with indicated antibodies. In each case loading control (actin) is from the same blot as the experimental sample. *p < 0.05; **p < 0.01. N=4–5 mice/group using Mann-Whitney test (**f-g**).

Given these findings, we sought to determine whether plasma EVs from aged, injured mice at 24h after injury had the potential to generate an inflammatory response in the CNS different cell types. Primary microglia, astrocytes, and neurons were cultured from mouse neonatal or embryonic cortices. Plasma EVs were isolated from young adult or aged mice at 1 d post-injury or sham mice. NTA was measured to obtain EVs concentration (EVs particles/ml). Based on pilot data, primary microglia were exposed to three different dosages (2 × 10^9^, 4 × 10^9^, and 6 × 10^9^ particles/ml) of EVs for 24 h, separately and the resulting supernatants were collected for ELISA assays. We initially assessed the secretion of cytokines CXCL2, IL-6, and TNFα in cultured microglia and found that CXCL2 release was increased in response to the EVs stimulation. In contrast, TNFα and IL-6 were barely detected in the microglial supernatants. EVs from young SCI-mice increased CXCL2 secretion in a dose-dependent manner compared to EVs stimulation from sham animals or PBS treatment (Fig. 9a). The highest concentration of EVs (6 × 10^9^ particles/ml) derived from young SCI mice significantly increased secretion of CXCL2 compared to Sham EVs or PBS (Fig. 9a, b). As SCI in aged mice causes profound neuroinflammation and neurodegeneration in the brain, we hypothesized that plasma EVs derived from these animals had more proinflammatory features contributing to the process. Therefore, we selected average EVs concentration (4 × 10^9^ particles/ml) from aged animals for microglial stimulation. EVs from aged SCI mice at a middle concentration level induced significant CXCL2 secretion (Fig. 9c, Fig. S4a-f). To ascertain the microglial responses were due to the EVs stimulation, we tested PBS, plasma supernatant collected after the ultracentrifugation spin. Fig. S4a-f showed that none of these were able to induce CXCL2 secretion in cultured microglia, indicating the specific effects of EVs derived from SCI animals.

Among these cytokines tested in cultured microglia, we found that IL-6 response to the EVs stimulation was marked in cultured astrocytes. Both EVs derived from young SCI mice at 6 × 10^9^ particles/ml or aged SCI animals at 4 × 10^9^ particles/ml significantly increased IL-6 secretion compared to their Sham EVs or PBS (Fig. 9d, e). We also tested PBS, plasma supernatant collected after the ultracentrifugation spin. Fig. S4g-l showed that none of these induced IL-6 secretion in cultured astrocytes, indicating the specific effects of EVs derived from SCI animals.

Primary cortical neurons were incubated with EVs derived from young or aged SCI mice. After 24 h treatment, the cells were harvested for protein extraction and neuronal apoptosis assay. Western blotting analysis showed that protein expression level of cleaved caspase 3 was significantly elevated in EV groups derived from young SCI mice at 6 × 10^9^ particles/ml or aged SCI animals at 4 × 10^9^ particles/ml (Fig. 9f–i). Fig. S5 showed additional experiments in which the EVs were derived from a different set of animals and the treatment in a different culture. Bafilomycin A1 (BFA, 100 nM) was used as a positive control for inducing neuronal apoptosis (Fig. S5). Together, these data indicate the pro-inflammatory features of circulating EVs from SCI mice, with greater effects of EVs-derived from aged SCI animals.

## Discussion

Despite the increasing incidence of SCI in older individuals, there have been relatively few experimental studies of SCI in aged animals, especially those examining posttraumatic brain changes. In the present study, we investigated the pathological changes at both the injury site and brain of young and aged mice following SCI, as well as potentially underlying cellular and molecular mechanisms. Aged SCI mice show worse neurological function, including motor, cognitive, and depressive-like behaviors. These changes are associated with increased tissue damage in injured spinal cord and exaggerated microglia responses in the brain. Our data also indicate that age-related deficits in autophagy are exacerbated in the brain following SCI. In addition, circulating EV and their miRs cargo content after SCI are more pronounced in aged animals.

Prior studies reported that aged spinal cord is more susceptible to traumatic injury in both mice and humans [20, 46]. Consistent with these results, we observed exacerbated tissue damage in aged mice following SCI. Previous work has shown that injury to the thoracic spinal cord causes profound neuropathological changes in the brain in young adult animals [29, 32, 33]. A recent study examining gene expression patterns in the brain after acute and sub-acute SCI, using transcriptome analysis with RNA sequencing, revealed that mitochondria dysfunction occurred at 3h post-injury, followed by increased inflammatory response and ER stress at 2 weeks after injury [3]. However, the brain changes following SCI in old age are largely unclear. In the present study, by utilizing a NanoString neuropathology panel directly targeting specific mRNAs, we unequivocally showed that SCI in old and young adult mice induce distinct transcriptomic profiles in the brain at 2 months post-injury. Comparisons between young and aged sham mice indicated genetic heterogeneity between cortex and hippocampus during aging. This is in agreement with a recent report that summarized the cellular and molecular heterogeneity of astrocytes and microglia in different brain regions in relation to aging and neurodegenerative diseases [24]. Through heatmap clustering of the average z-scores for each group, our findings demonstrate that (1) the “Activated Microglia” and “Autophagy” are the top two upregulated pathways in the hippocampus, but not in the somatosensory cortex with aging, and (2) these pathways become pronounced following SCI in the cortex from aged but not young adult animals.

Brain and spinal cord aging lead to increased inflammatory activity, peripheral immune cell infiltration, and decreased autophagy efficiency. Previous work has found that cellular and molecular markers of inflammation in the injured spinal cord are acutely altered after SCI in aged animals [13, 60]. We observed similar changes in aged mice during the chronic phase of SCI, using a combination of ex vivo cellular assays and histological approaches. At the cellular level, increased microglial proliferation and infiltrating myeloid cells occurred at the injury site at 2 months in aged mice. Lymphocyte infiltration was also increased in old mice at this time-point. Similar changes have been reported in aged models of brain trauma and experimental stroke [11, 50], suggesting that this may be a conserved age-related response to CNS injury. These aged related cellular changes contribute to greater tissue damage at the injured site. In the brain, older mice showed fewer microglia compared to young adult animals, likely either due cell senescence and/or death, consistently with the previously reports [50, 69]. However, myeloid cells and lymphocyte infiltration were prominent in old mice, with increased lymphocyte recruitment after chronic injury. To ascertain whether the changes of microglia and leukocytes in the brain after SCI were affected in old age, we performed an extensive profiling of cellular functions. The most pronounced effects of age were microglial phagocytosis and dysregulated autophagy. In general, older microglia, especially in injured mice, were more likely to engulf neuronal debris or form autophagosome or lysosome than their younger counterparts. Moreover, we demonstrate perturbations in synaptic function and autophagy of aged neurons. These age-related alterations in inflammatory activity, phagocytosis, and autophagy at the cellular levels are consistent with the results from the NanoString analysis. It is known that brain aging leads to decline in autophagy efficiency, increasing the probability of protein aggregation and contributing to a higher prevalence of neurodegenerative diseases [42]. Our data indicate that age related decline in autophagy and lysosomal function in microglia and neurons in the mouse brain was exacerbated by SCI. This was accompanied by increased brain inflammation and neurodegeneration, suggesting that perturbation of autophagy may provide a mechanistic link between SCI and posttraumatic brain dysfunction.

We and others have shown that a microglial activator chemokine (C-C motif) ligand 21 (CCL21) produced by injured dorsal horn neurons is directly transported to the thalamus [68], and accumulates in cortex and hippocampus at sub-acute and chronic phases [64, 65], contributing to pro-inflammatory responses in the brain after SCI. Emerging data suggest that EVs may participate in the progression of secondary injury by transporting parent cell-specific signaling cargoes (e.g., signal lipids, genetic information, cytokines, receptors, etc.) that alter the function of recipient cells both within and outside the CNS [12, 53]. However, circulating EV-mediated crosstalk following SCI and its underlying molecular mechanism(s) has received limited attention. Recently, our lab reported that SCI in young adult mice potentiated the circulating EV response with altered miR cargo, contributing to upregulated expression of inflammation-related genes in the brain [22]. In the present study, we show that old age alters the EV response in both circulation and injured tissue, as well as miR cargo, following SCI. We observed 3 miRs (miR-125b-5p, miR-206, miR-145–5p) that are increased in plasma EVs, but decreased in spinal cord EVs after injury. miR-145–5p negatively affects cell proliferation and chemokine secretion, as well as acting as a regulator of SOX2 [45, 66]. miR-206 is enriched in mouse and human skeletal muscle and is critical for myogenesis [57, 58]. miR-125b-5p acts as a negative co-regulator of some inflammatory genes through the TRAF6/MAPKs/NF-κB pathway [47]. These miRs may shuttle from spinal cord to plasma after pro-inflammatory activation, serving as regulators that modify gene expression in downstream target cells. Moreover, in the injured tissue, we showed that let-7d-5p increased and miR-103a-3p decreased in aged SCI mice compared to young SCI animals. The former suppresses inflammatory responses, whereas the latter reduces apoptosis and inflammation by targeting HMGB1 [27, 59]. Thus, altered miR cargo after SCI impacts inflammation and autophagy related pathways, potentially contributing to subsequent neuropathology.

We also demonstrated that cultured mouse cortical microglia and astrocytes exhibit pro-inflammatory responses after exposure to SCI-derived blood-borne EVs, suggesting that plasma EVs are capable of transducing injury signals from damaged tissue to the brain following SCI. The enhanced expression of pro-inflammatory cytokines (CXCL2 and IL-6) and neuronal apoptotic marker (Caspase 3) further indicate the potential of injury induced EVs to potentiate inflammatory and cell death responses, whereas enhanced microglial phagocytosis may contribute to senescence-associated neurodegeneration [6]. Moreover, EVs from aged SCI mice caused pathophysiological changes *in vitro* at a lower concentration than those from young adult mice. Together with our findings from flow cytometry and transcriptomic data, these findings support the hypothesis that circulating EVs after SCI in aged brain may contribute to increased neuroinflammatory and neurodegenerative responses, resulting in worse neurological outcome.

In summary, our histological, cellular, and molecular findings provide complementary evidence that SCI in aged mice increases pathophysiological responses involving microglial activation and the autophagy-lysosome pathway in the brain, exacerbating neurodegeneration and neurological dysfunction. We also report pro-inflammatory features of circulating EVs derived from aged animals following SCI that may contribute to glia-mediated inflammation and neuronal cell death. The EVs released following SCI may also affect outcome by affecting the well-known systemic effects of SCI.

## Data Availability

All data needed to evaluate the conclusions in the paper are present in the paper and/or the Supplementary Materials.
